# Supplementary material for: Lipid order and charge protect killer T cells from accidental death
Source: Nat Commun. 2019 Nov 27;10:5396. doi: 10.1038/s41467-019-13385-x (PMC6881447; doi:10.1038/s41467-019-13385-x)
Supplement: Supplementary file 1 — Supplementary Information [file 41467_2019_13385_MOESM1_ESM.pdf]

Lipid order and charge protect killer T cells from accidental death

Supplementary Information

Jesse A. Rudd-Schmidt, Adrian W. Hodel, Tahereh Noori, Jamie A. Lopez, Hyun-Jung Cho, Sandra Verschoor, Annette Ciccone, Joseph A. Trapani, Bart W. Hoogenboom & Ilia Voskoboinik

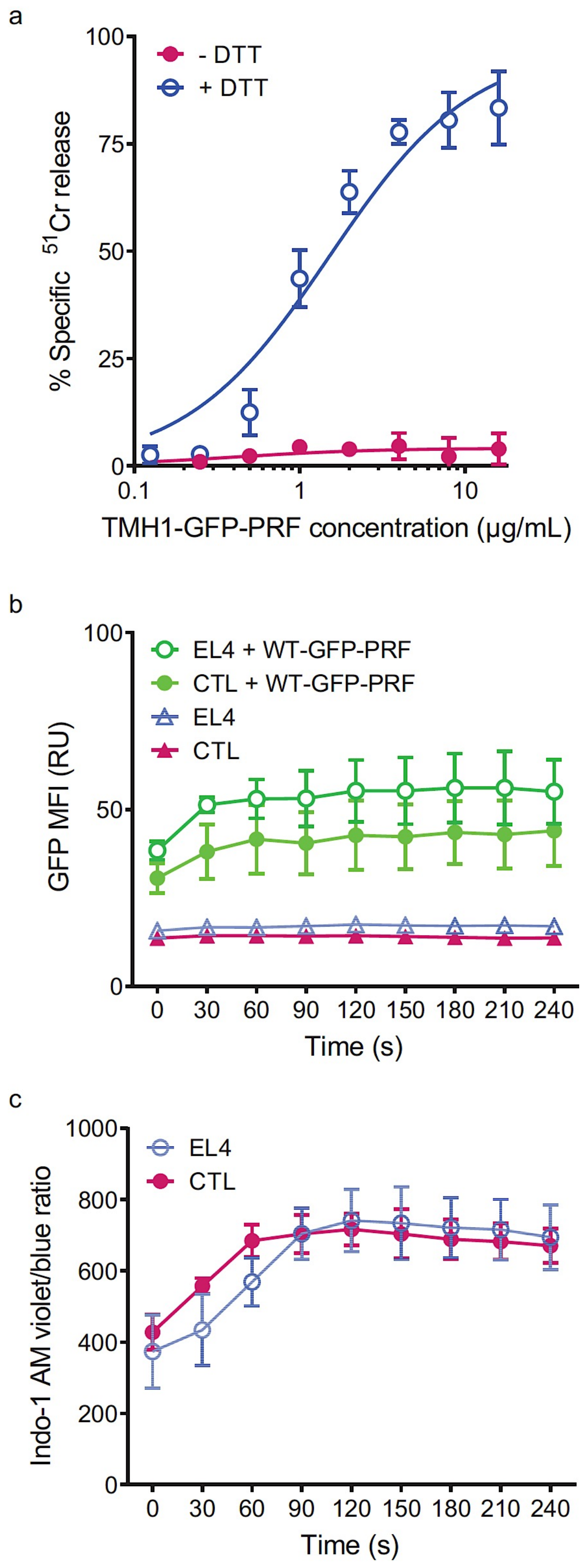

Supplementary Figure 1. Control experiments validating TMH1-GFP-PRF mutant and Ca^{2+} flux measurements.

a, Functionality of TMH1-GFP-PRF as assessed by ^{51}Cr release assay. Recombinant TMH1-GFP-PRF is not cytotoxic to EL4 cells over a wide range of perforin concentrations (-DTT). However,

when 0.75 mM DTT is added to this cell-bound perforin, the engineered disulphide-bond in TMH1-GFP-PRF is unlocked. As a consequence, the functionality of TMH1-GFP-PRF is restored, as here demonstrated by its cytotoxicity to the EL4 cells (^{51}Cr release increasing with TMH1-GFP-PRF concentration, +DTT). Each data point represents a mean (± standard deviation, SD) of 3 independent experiments; curves represent Michaelis-Menten fits to the data. For an extensive validation of the behaviour and functionality (+DTT) of the TMH1-PRF mutant see [1].

b, WT-GFP-PRF was dosed such that similar amounts bound to the EL4 cells and CTLs. To this end, firstly a sub-lytic amount of WT-GFP-PRF was determined for the EL4 cells (on the day of each experiment), and then increasing amounts of WT-GFP-PRF were added to the CTLs until a similar MFI was achieved, and experiments were then conducted with these optimized conditions. Each data point represents a mean (± SD) of 3 independent experiments. An unpaired t-test performed on EL4 + WT-GFP-PRF and CTL +WT-GFP-PRF shows $P<0.05$ for $t = 30$ s, but no significant difference for all other time points.

c, Indo-1 AM violet/blue fluorescence ratio (400 nm/475 nm) increases in both cell types when Ca^{2+} influx is induced by the Ca^{2+} ionophore ionomycin, demonstrating that both types of cells show a similar response to Indo-1 AM labelling. Each data point represents a mean (± SD) of 3 independent experiments.

Source data for all panels are provided as a Source Data file.

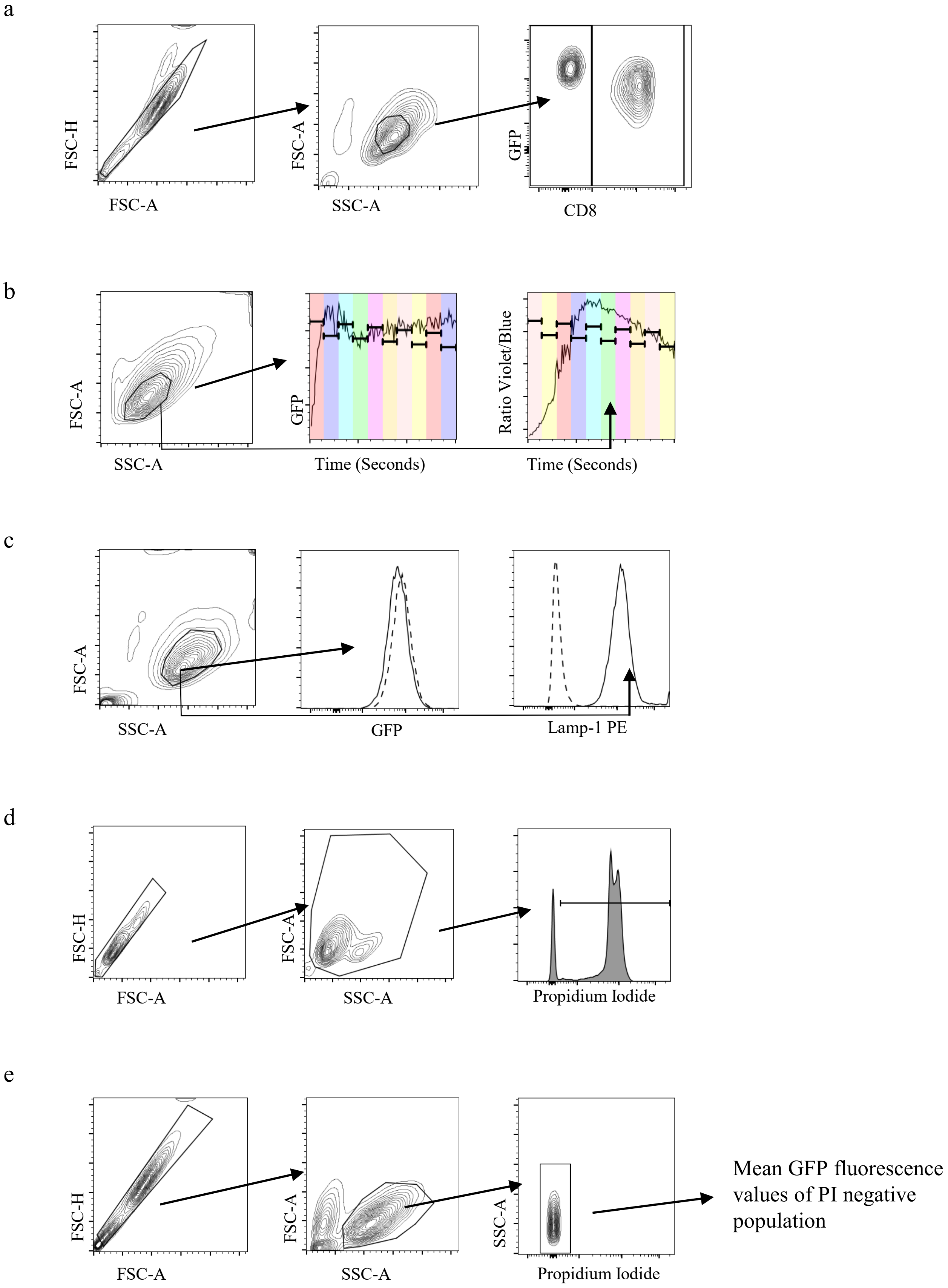

Supplementary Figure 2. Gating Strategies used for flow cytometry analysis.

a, Gating strategy used to analyse cells of the same size within a mixture of CD8+ T cells and EL4 target cells for either perforin vs CD8 (Figs. 1b, 2a,b,c) or GM1 vs CD8 (Supplementary Fig. 8).

b, Gating strategy used to analyse EL4 or CD8+ T cells of the same size for perforin binding over time (Supplementary Fig. 1b) and Indo-1 AM fluorescence (Violet/Blue) over time (Fig. 1c, Supplementary Fig. 1c).

c, Gating strategy used to analyse equal GFP expression of EL4 and CD8+ T cells transduced with either empty MSCV-GFP (dashed line) or truncated LAMP-1-MSCV-GFP (solid line) (Supplementary Fig. 3), and surface LAMP-1 expression (as detected by anti-LAMP1-PE antibodies) of EL4 or CD8+ T cells transduced with truncated LAMP-1.

d, Gating strategy used to analyse cell death (propidium iodide positivity) of EL4 or CD8+T cells treated with 7KC (Fig. 4b) or with 7KC and perforin (Fig. 4e).

e, Gating strategy used to analyse the mean GFP fluorescence of CD8+ T cells treated with 7KC and then incubated with TMH-GFP perforin (Fig. 4d).

a

b

c

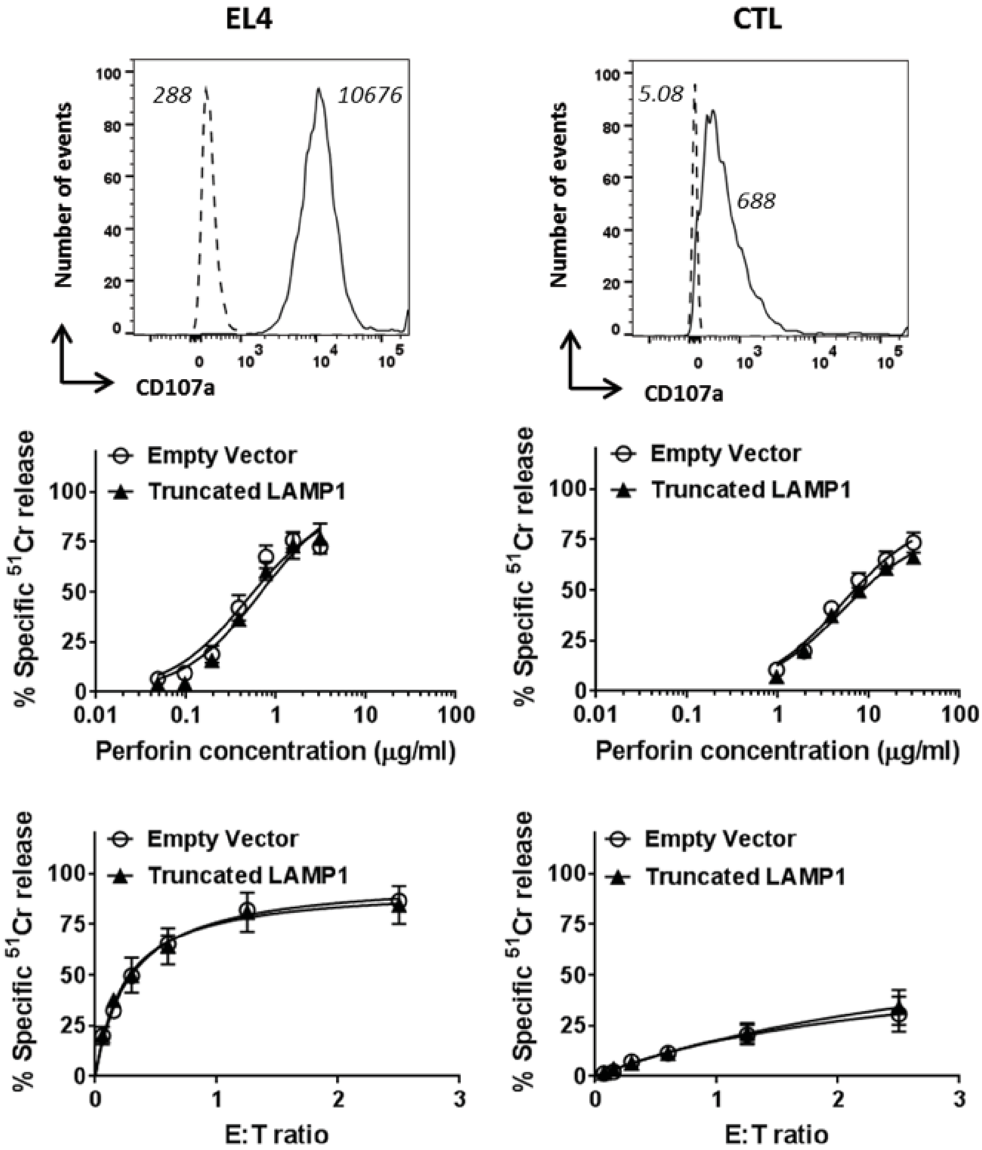

Supplementary Figure 3. High surface levels of CD107a (LAMP-1) do not protect cells from recombinant perforin lysis or killing by CTLs.

a, Surface staining for CD107a of EL4 and *Prf1*$^{-/-}$ OTI (CTLs). EL4 cells (left) transduced with a truncated form of CD107a [2] (shown by solid line) have a 37-fold higher surface expression of CD107a than EL4 cells transduced with an empty vector (shown by dashed line), as detected by surface staining with anti-CD107a-Phycoerythrin antibody. CTLs (right) transduced with the same truncated form of CD107a (shown by solid line) have a 135-fold higher surface expression of CD107a than CTL cells transduced with an empty vector (shown by dashed line). Both histograms are representative examples of surface staining performed on the day of every experiment, the number of events has been standardized to the mode to allow clear comparison of both histograms and geometric mean fluorescence intensity (MFI) values from which fold difference values were calculated are shown next to each peak.

b, ^{51}Cr release of EL4 (left) and CTL (right) cells upon exposure to recombinant WT-PRF. There is no significant difference in perforin sensitivity between cells transduced with an empty vector and with truncated CD107a.

c, ^{51}Cr release of SIINFEKL labelled EL4 (left) and CTL (right) target cells incubated with activated WT OTI CTLs, as a function of the ratio of effector to target cells (E:T ratio). Again, there is no significant difference in sensitivity to killing between target cells transduced with an empty vector or with truncated CD107a, for both EL4 and CTL target cells.

For **b,** and **c,** each data point represents a mean (± standard error of mean) of 3 independent experiments; curves represent Michaelis-Menten fits to the data. Source data for these two panels are provided as a Source Data file.

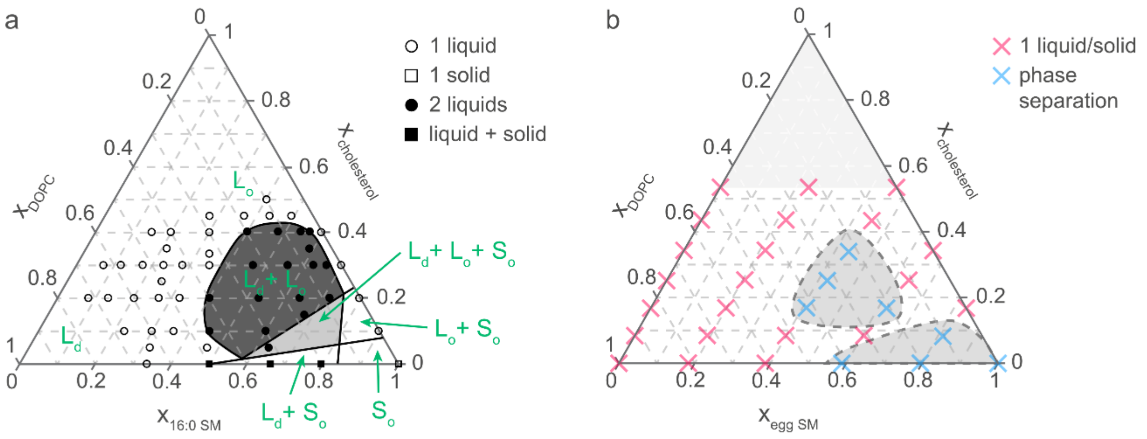

Supplementary Figure 4. Phases and phase coexistence in ternary DOPC/SM/cholesterol lipid systems.

a, Map of DOPC/16:0 SM/cholesterol mixtures, and the lipid phases in giant unilamellar vesicles (adapted from Veatch and Keller 2005 [3], with information from Marsh 2013 [4]). Mixtures in the bottom left corner (DOPC-rich) are in the L_d state. Membrane order increases by addition of cholesterol (L_o in the top regions) or 16:0 SM (S_o in the right corner). Phase separation is observed in mixtures located in the lower right side of the triangular map.

b, Map of DOPC/egg SM/cholesterol mixtures as used for our experiments. Mixtures that show phase separation – detected via the appearance of domains with distinct membrane thicknesses – in our experiments are marked with a blue “x”, and the remaining, single-phase compositions with a red “x”. The phase separated mixtures are encircled by a dashed line and further highlighted with a grey background. The areas that show phase separation are similar in the overview based on literature values **(a)** and in our own experiments in **(b)**.

Minor difference may arise because, in contrast to the 16:0 SM used in **a,** the egg SM used in b is a mixture of different SM species (though mostly 16:0 SM as per supplier specifications) that inherently shows phase separation. Also note that we tested mixtures containing up to 53% cholesterol. Above ca. 66% in PC/cholesterol mixtures, cholesterol is known to crystallize. The data in both maps was recorded at 37 °C.

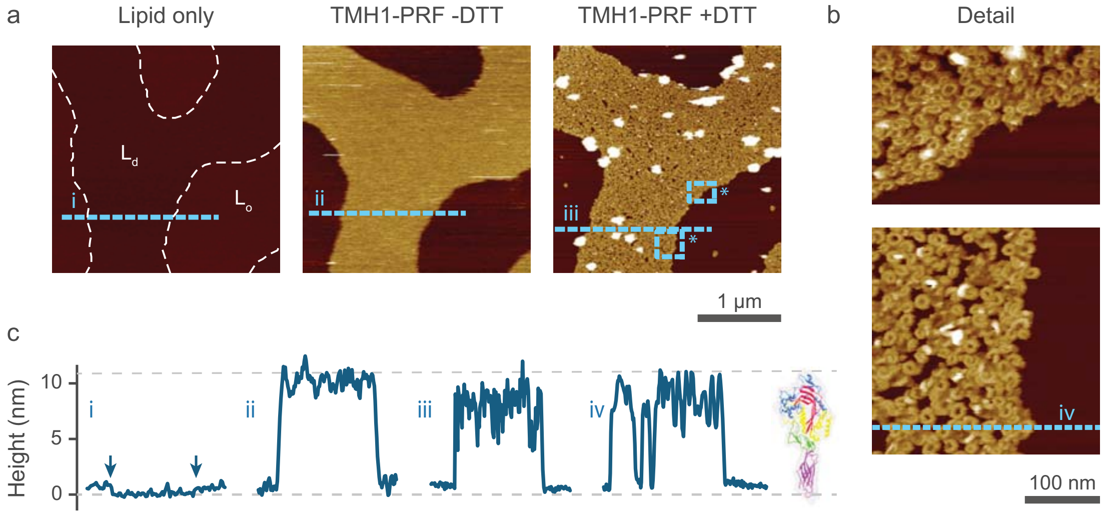

Supplementary Figure 5. Prepore-locked TMH1-PRF binds to disordered domains on phase separated lipid membranes.

a, AFM images of an approximately equimolar DOPC/egg SM/cholesterol supported lipid bilayer, showing phase separation between liquid disordered (L_d) and liquid ordered (L_o) domains, with the L_o phase appearing higher (thicker) than the L_d phase in the "lipid only" image. The phase boundaries are highlighted by dashed white lines. Prepore-locked perforin (TMH1-PRF -DTT) appears as diffuse plateaus due to its mobile nature [1], exclusively bound to the L_d domains. Upon exposure to DTT, these mobile prepores transform into static pores (TMH1-PRF +DTT).

b, Higher-magnification images of the areas indicated by the dashed blue rectangles in **a**. The L_d domain shows a dense coverage of arc- and ring-shaped pores, whereas the L_o domains remains empty.

c, Height profiles as recorded along the blue, dashed lines in the AFM images in **a** and **b**. The phase separation on the empty membrane (i) is visible as a 0.5-1 nm height difference, and the phase boundaries are indicated by vertical arrows. Addition of TMH1-PRF (-DTT/+DTT, ii-iv) leads to the formation of ca. 11 nm high features, corresponding to the height of perforin prepores and pores [1,5,6]. Grey, dashed lines indicate the height of the membrane (0 nm) and of a perforin monomer (11 nm [5]). The differences in measured perforin height between traces ii, iii and iv are attributed to variations in the applied forces in the AFM experiments. The sample was incubated and imaged at 37 °C. Colour (height) scale as in Fig. 3b.

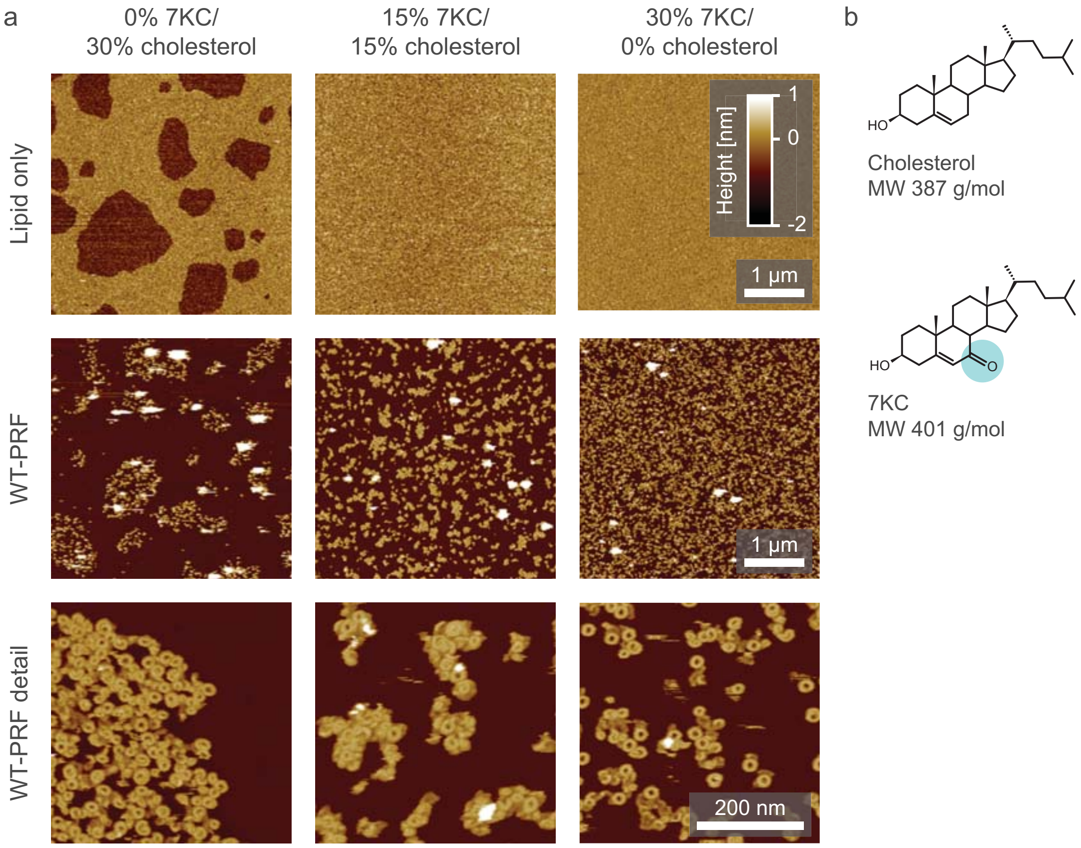

Supplementary Figure 6. Effect of 7KC on membrane order, as assessed by AFM.

a, AFM images of DOPC/egg SM/sterol 20:50:30 supported lipid bilayers, as a function of sterol content (7KC, cholesterol). The images show membranes before and after exposure to 150 nM WT-PRF. Note that in the top row, the colour scale was enhanced to better show phase separation, while the middle and bottom rows use the same colour scale as Fig. 3b-d. For 0% 7KC / 30% cholesterol, these lipids phase-separate into L_d and L_o domains (respective dark and bright areas in the "lipid only" image, see also Fig. 3a,b and Supplementary Fig. 4). Exposure to WT-PRF leads to pore formation exclusively in L_d domains, consistent with Fig. 3 and Supplementary Fig. 4. For 15% 7KC / 15% cholesterol, no phase separation is observed at micrometre length scales, and perforin pores are formed more uniformly over the sample surface. At nanometre length scales (bottom row), some phase separation persists, here visible by the clustering of perforin pores (presumably bound to nanometre-scale L_d domains). For 30% 7KC / 0% cholesterol, perforin binds uniformly over the whole sample surface without noticeable pore clustering in domains.

b, Chemical structures and molecular weights for cholesterol and 7KC, with the additional ketone group highlighted. All samples were incubated and imaged at 37 °C.

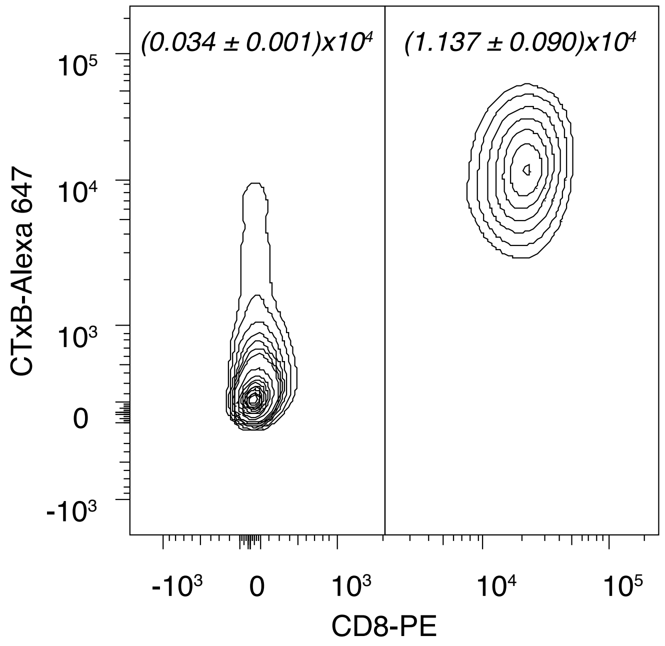

Supplementary Figure 7. GM1 surface staining of CTL and EL4 cells by cholera toxin subunit B (CTxB) Alexa 647.

GM1 surface staining by recombinant cholera toxin subunit B Alexa 647. GM1 intensity (represented by CTxB) has been plotted against $CD8^+$ positivity to identify $CD8^+$ T cells in a 1:1 mixture of EL4 and OTI T cells (cells are gated for same size as detailed for perforin binding experiments in manuscript). Average MFI (± SD) of CTxB-Alexa 647 from 3 independent experiments is included for both CD8 positive and negative cells and provided as a Source Data file.

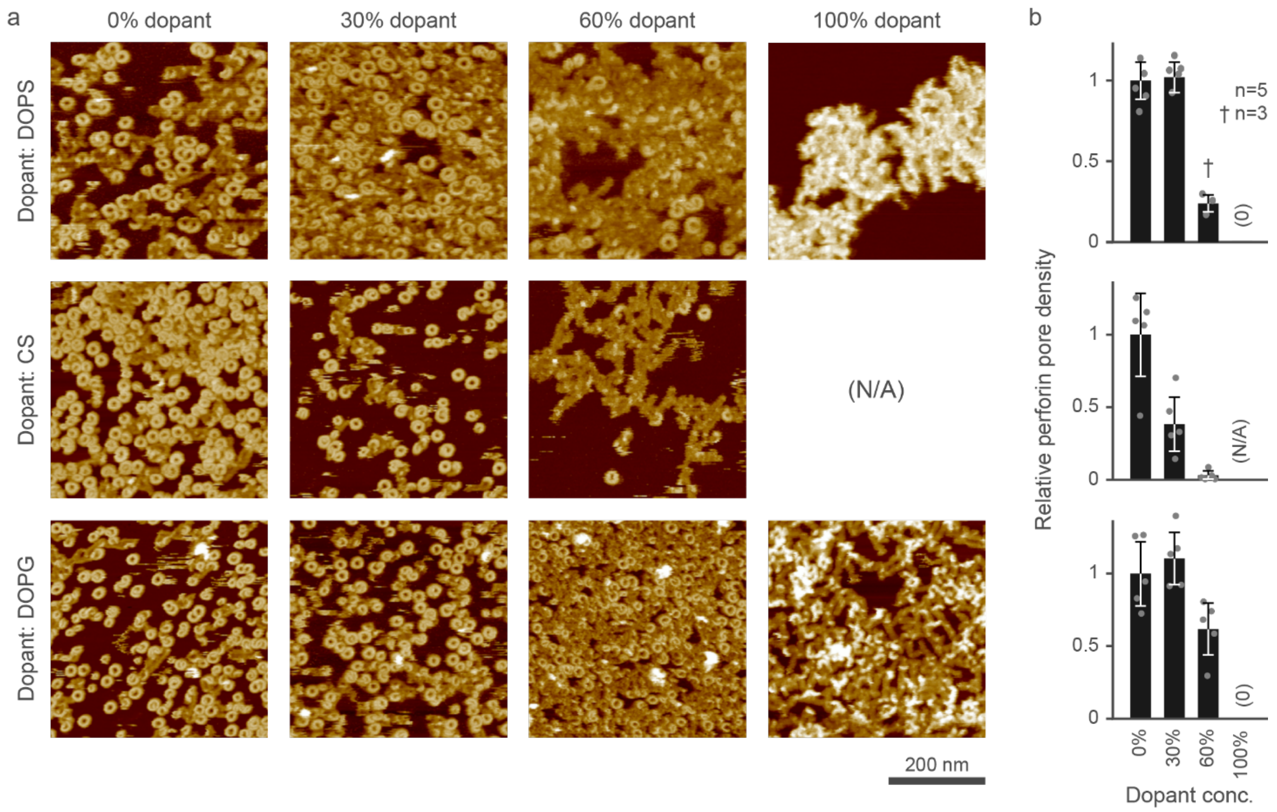

Supplementary Figure 8. Perforin forms non-porating clusters on the negatively charged membranes.

Perforin pore formation on DOPC model membranes doped with the negatively charged membrane components DOPS, DOPG (1,2-dioleoyl-sn-glycero-3-phospho-(1'-racglycerol)), and CS (cholesterol sulfate), as assessed by AFM.

a, Representative images of the sample surfaces of samples containing 0%, 30%, 60% and 100% of either DOPS, DOPG, or CS in DOPC host membranes (for 100% CS, no supported lipid bilayers could be formed). Colour (height) scale as in Fig. 3b.

b, Quantification of pore formation (mean ± SD) in the samples shown in a, relative to the 0% dopant/ 100% DOPC reference. The data on DOPS was reproduced from Fig. 5b, for comparison. Source data are provided as a Source Data file.

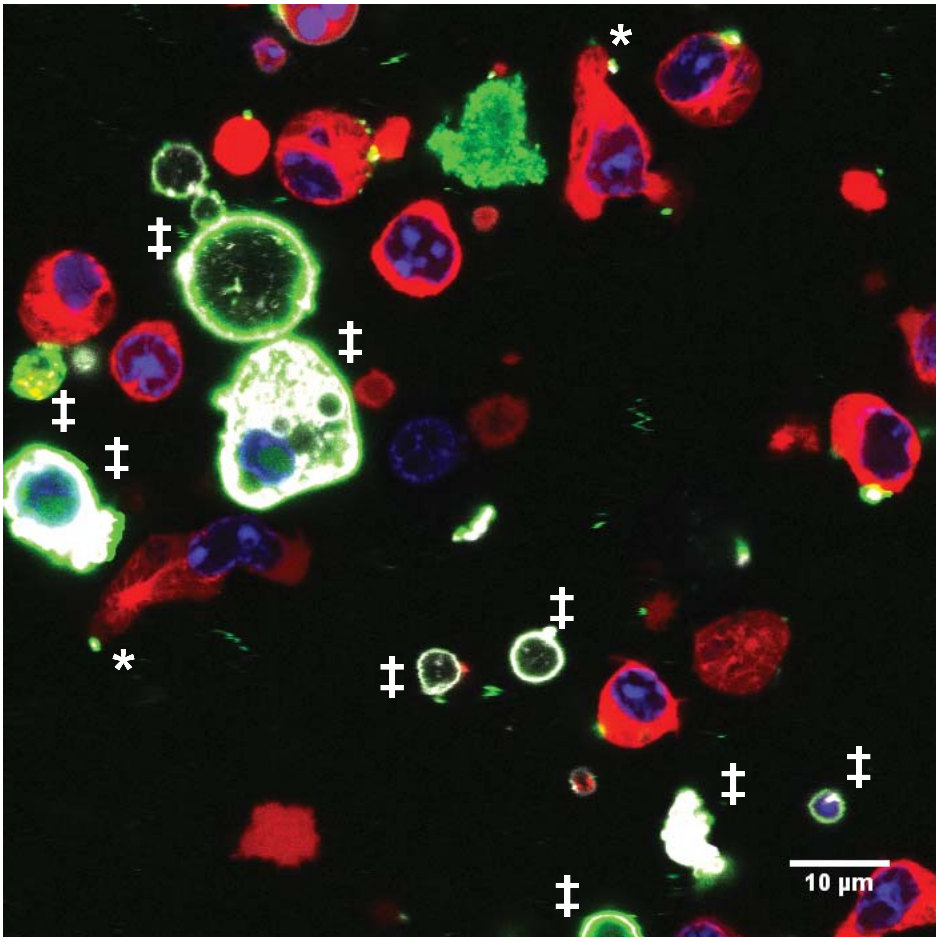

Supplementary Figure 9. Unlike target cells, CTL maintain healthy morphological appearance and mobility one hour after addition of WT-GFP-PRF.

CTLs (red) with clear WT-GFP-PRF signal (green) localized to punctate regions of non-apoptotic PS (white) 1 hour after addition of perforin. Dead target cells and their debris are stained bright green and white (highlighted by white daggers), because of, respectively, their overall WT-GFP-PRF binding and staining for apoptotic PS. Note that on polarized CTLs, non-apoptotic PS is always located within the uropod (white asterisks). Staining is as in Fig. 7c; the data shown here represent a snapshot from Supplementary Video 2.

Supplementary References

1. Leung, C. *et al.* Real-time visualization of perforin nanopore assembly. *Nat. Nanotechnol.* **12**, 467–473 (2017).
2. Cohnen, A. *et al.* Surface CD107a / LAMP-1 protects natural killer cells from degranulation-associated damage. *Blood* **122**, 1411–1418 (2013).
3. Veatch, S. L. & Keller, S. L. Miscibility Phase Diagrams of Giant Vesicles Containing Sphingomyelin. *Phys. Rev. Lett.* **94**, 148101 (2005).
4. Marsh, D. *Hanbook of lipid bilayers*. (CRC Press, 2013).
5. Law, R. H. P. *et al.* The structural basis for membrane binding and pore formation by lymphocyte perforin. *Nature* **468**, 447–51 (2010).
6. Metkar, S. S. *et al.* Perforin oligomers form arcs in cellular membranes: a locus for intracellular delivery of granzymes. *Cell Death Differ.* **22**, 74–85 (2015).
