# Supplementary material for: Lipid order and charge protect killer T cells from accidental death
Source: Nat Commun. 2019 Nov 27;10:5396. doi: 10.1038/s41467-019-13385-x (PMC6881447; doi:10.1038/s41467-019-13385-x)
Supplement: Supplementary file 6 — Description of Additional Supplementary Files [file 41467_2019_13385_MOESM6_ESM.pdf]

Title: Supplementary Video 1.
Description: Recombinant WT-GFP-PRF binds uniformly to and lyses EL4 cells, but on CTLs it localizes to distinct regions of exposed phosphatidylserine. WT-GFP-PRF (shown in green, added at time point 1:17 min) was added to a mixture of CTLs transduced with cherry-tubulin (shown in red) and EL4 cells. Hoechst 33342 (shown in blue) staining shows the nucleus of both cell types. Annexin V Alexa 647 (shown in white) was maintained in the culture medium throughout the assay; it binds to non-apoptotic phosphatidylserine exposed on the CTLs and to PS on and within EL4 cells that are exposed to cytotoxic levels of perforin and lose membrane integrity. EL4 cells are seen to bind WT-GFP-PRF uniformly before gradually becoming annexin V positive, whilst perforin on the CTL membrane localizes precisely (and almost immediately) to regions of exposed PS, without lysing the cells.

Title: Supplementary Video 2.
Description: Unlike target cells, CTL maintain healthy morphological appearance and mobility one hour after addition of WT-GFP-PRF. CTL (red) are seen to move across the field of view, with clear WT-GFP-PRF signal (green) localized to punctate regions of exposed PS (white), 1 hour after addition of perforin (here defined as t = 0 min). Dead target cells and their debris are stained bright green and white (highlighted by white daggers), because of, respectively, their overall WT-GFP-PRF binding and staining for apoptotic PS. Staining is as in Fig. 7c and Supplementary Video 1.

Title: Supplementary Video 3.
Description: Phosphatidylserine is exposed on the CTL membrane during synapse formation. Time-lapse confocal microscopy reveals a bright punctate region of PS exposed on the Prf1-/- CTL membrane (detected by annexin V Alexa Fluor 488 maintained during assay; shown in green) during synapse formation. SIINFEKL labelled target cells (MC57) were labelled with Cell Trace Violet (shown in blue) and synapse formation is confirmed by the docking of the centrosome (detected by cherrytubulin expressed in the CTL; shown in red) at the point of contact between CTL and target.
